# Repair Bond Strength of Ion-Releasing Versus Conventional Resin Composites

**DOI:** 10.3390/ma19061076

**Published:** 2026-03-11

**Authors:** Jenny Buhl, Matej Par, Andrea Gubler, Tobias T. Tauböck

**Affiliations:** 1 Department of Conservative and Preventive Dentistry, Center for Dental Medicine, University of Zurich, 8032 Zurich, Switzerland; jenny.buhl@zzm.uzh.ch (J.B.); andrea.gubler@zzm.uzh.ch (A.G.); 2 Department of Endodontics and Restorative Dentistry, University of Zagreb School of Dental Medicine, 10000 Zagreb, Croatia; mpar@sfzg.unizg.hr

**Keywords:** ion-releasing resin composite, bioactive glass filler, resin composite, micro-tensile bond strength, repair bond strength, failure analysis

## Abstract

With the growing clinical use of ion-releasing resin composites, their repairability has become an important consideration in minimally invasive restorative dentistry. Therefore, this study investigated the repair bond strength of a universal composite restorative to commercially available and experimental ion-releasing resin composite materials. Specimens (*n* = 8 per group) were produced from three commercially available ion-releasing composite materials (ACTIVA BioACTIVE-RESTORATIVE, Cention Forte, Beautifil II), one experimental ion-releasing resin composite containing 20 wt% bioactive glass fillers, and two conventional resin composites (3M Filtek Supreme XTE, Ceram.x Spectra ST), and aged by thermal cycling in artificial saliva (5000 cycles, 5–55 °C). Substrate surfaces were sandblasted (Al_2_O_3_, 50 µm), silanized (Monobond Plus), and repaired using adhesive (OptiBond FL) and universal resin composite (Ceram.x Spectra ST). After further thermal cycling, micro-tensile repair bond strength was assessed and analyzed using one-way ANOVA followed by Tukey’s post hoc test. Failure modes were determined by stereomicroscopy (25× magnification) and statistically compared among the groups. Highest mean repair bond strength values were obtained for ACTIVA BioACTIVE-RESTORATIVE, Beautifil II, and 3M Filtek Supreme XTE (53.8, 46.2, and 43.0 MPa, respectively), which did not differ significantly among each other. ACTIVA BioACTIVE-RESTORATIVE attained significantly higher bond strength than the experimental composite, Ceram.x Spectra ST, and Cention Forte, and showed the highest incidence of cohesive failures (40%). No significant bond strength differences were detected among Beautifil II, 3M Filtek Supreme XTE, experimental composite, Ceram.x Spectra ST, and Cention Forte (36.2–46.2 MPa). In conclusion, ion-releasing resin composites can be repaired with conventional universal composite and show repair bond strength values at least as high as those of conventional composite materials.

## 1. Introduction

Secondary caries serves as the primary reason for the replacement of dental restorations [1,2,3]. Specifically, in the case of resin-based composite restorations, the material’s polymerization-induced shrinkage generates stresses at bonded restoration margins [4,5], which can lead to the development of marginal gaps between the restorative material and tooth tissue. These gaps may contribute to postoperative sensitivity [6,7], marginal discoloration [8], and secondary caries [9].

One approach to addressing the issue of secondary caries is the incorporation of bioactive glass as additional fillers into dental composites. The core principle involves enhancing well-established dental composites by modifying them with bioactive fillers, resulting in a therapeutic restorative material with remineralizing and antibacterial properties. These bioactive fillers encompass glass compositions embedded in an SiO_2_–P_2_O_5_–CaO–Na_2_O system [10]. The favorable outcomes are achieved through the release of Ca^2+^, PO_4_^3−^, and F^−^ ions [10]. The objective of bioactive glasses is to induce the formation of an apatite phase within the marginal gap, providing a marginal seal and mitigating acid attacks by elevating the local pH [11].

Despite their potential advantages in preventing secondary caries, most ion-releasing composites generally exhibit inferior mechanical properties compared with conventional resin composites [12,13]. Degradation of the matrix, as well as partial dissolution of reactive filler particles occur, particularly after aging [14,15].

After thermocycling, a decrease in fracture toughness has been observed [16]. While the wear mechanisms of ion-releasing composites resemble those of conventional resin composites following aging, ion-releasing materials show a higher incidence of fractures and increased porosity [16]. The flexural modulus of ion-releasing composites, ideally matching dentin (11.4–25.0 GPa) [17,18,19], is often lower, especially under varying pH conditions [17].

Given their susceptibility to fractures, repair strategies are of particular interest in order to extend the lifespan of ion-releasing composite restorations. Awad et al. [20] investigated the repair bond strength of the bioactive restorative material ACTIVA BioACTIVE-Restorative, focusing primarily on different repair protocols and found that air abrasion positively affected the repair bond strength of this bioactive restorative material. Ozaslan et al. [21] conducted a comparative study evaluating the reparability of two ion-releasing materials (Cention N and Equia Forte HT Fil) in comparison with Filtek Z550. They concluded that the repair bond strength of the conventional resin composite group was the highest, while the ion-releasing materials exhibited similar bond strength values among each other.

However, the number of ion-releasing resin-based composites available on the market has increased considerably in recent years, and many of these materials may require repair in clinical practice. Therefore, the objective of the present in vitro study was to investigate the repair bond strength of a universal composite restorative to commercially available and experimental ion-releasing resin composite materials. The null hypothesis tested was that there are no significant differences in micro-tensile repair bond strength between ion-releasing composites (ACTIVA BioACTIVE-RESTORATIVE, Beautifil II, Cention Forte, experimental composite) and conventional resin composites (Ceram.x Spectra ST, 3M Filtek Supreme XTE) under the applied aging and surface treatment conditions.

## 2. Materials and Methods

### 2.1. Composite Materials

The study included three ion-releasing composites (ACTIVA BioACTIVE-RESTORATIVE (Activa), Pulpdent, Watertown, MA, USA; Beautifil II (Beautifil), SHOFU, Kyoto, Japan; Cention Forte (Cention), Ivoclar, Schaan, Liechtenstein), one experimental ion-releasing resin composite (Experimental) containing 20 wt% bioactive glass fillers, and two conventional resin composites (Ceram.x Spectra ST (Ceram), Dentsply Sirona, Konstanz, Germany; 3M Filtek Supreme XTE (Filtek), 3M Oral Care, St. Paul, MN, USA).

The experimental composite was formulated according to Par et al. [22] using a photocurable resin system consisting of bisphenol-A-glycidyldimethacrylate (Bis-GMA) and triethylene glycol dimethacrylate (TEGDMA) in a 60:40 wt% ratio. The photoinitiator system consisted of camphorquinone (0.2 wt%) and ethyl-4-(dimethylamino)benzoate (0.8 wt%). Those components were mixed on a magnetic stirrer (IKAMAG RCT, IKA, Staufen, Germany) in a dark bottle to prevent premature polymerization. The experimental composite was prepared by mixing the photocurable resin with the experimental low-Na F-containing bioactive glass using a speed mixer (FlackTek Speed Mixer DAC 330-100 SE, FlackTek Inc., Louisville, CO, USA) at 2000 rpm for 15 min, followed by dark storage under vacuum conditions. The exact composition of the low-Na F-containing bioactive glass prepared on-demand by Schott (Mainz, Germany) via a melt-quench route is provided in Table 1.

**Figure 1:**
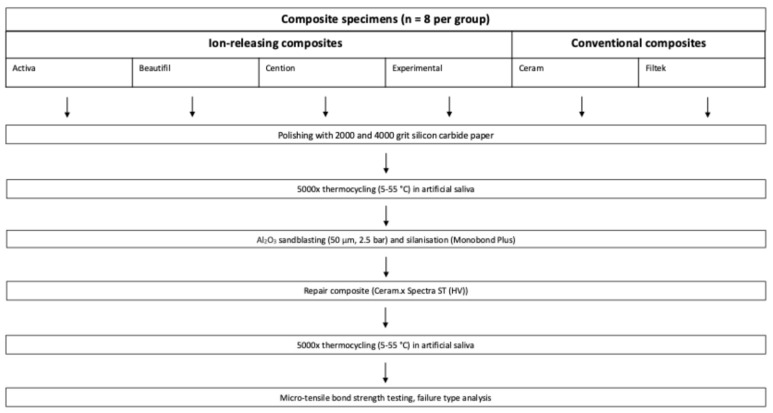
Experimental design of the present study.

For a comprehensive view of the experimental setup, including the different material groups, refer to Figure 1. Further details regarding the chemical composition and additional information about the resin composite materials utilized in this study can be found in Table 1.

### 2.2. Specimen Preparation

Eight specimens of each composite material were fabricated. Specimens of Activa, Beautifil, Experimental, Filtek, and Ceram were prepared by layering three composite increments, each measuring 1.5 mm in thickness, on scanning electron microscope (SEM) carriers (Wenka, Karl Wenger SA, Courgenay, Switzerland) using custom-made cylindrical Teflon molds with a diameter of 10 mm. All commercial composites were in shade A2. To ensure a flat surface, conventional modeling instruments were employed. Each increment was light-cured for 20 s at an irradiance of 1200 mW/cm^2^ (Bluephase G2, Ivoclar, Schaan, Liechtenstein). The distance between the specimen surface and the light-curing unit was kept constant by placing the tip of the light-curing unit onto a 1 mm thick glass plate. The radiant exitance was periodically verified using a calibrated dental radiometer (FieldMaxII-TO, Coherent; Santa Clara, CA, USA).

For fabrication of the dual-curing Cention-specimens, one large increment with 4.5 mm thickness was placed on the SEM carriers using a custom-made cylindrical Teflon mold (diameter 10 mm) and light-cured for 20 s at an irradiance of 1200 mW/cm^2^ (Bluephase G2, Ivoclar, Schaan, Liechtenstein).

The composite specimens were polished under constant water cooling using 2000- and 4000-grit silicon carbide (SiC) paper (Buehler-Met II, Buehler, Esslingen, Germany) in a polishing device (Tegramin-30, Struers, Ballerup, Denmark). Subsequently, the specimens were submerged in baths of artificial saliva (prepared according to the formulation of Cho et al. [23]), and artificially aged using a thermocycling machine (Haake W15, Thermo, Willytec, Gräfeling, Germany). A total of 5000 thermal cycles (5–55 °C; dwell time: 20 s in each bath; transfer time: 10 s) were completed, as described by Wiegand et al. [24] (dwell time: 20 s in each bath; transfer time: 10 s; duration of each cycle: 50 s).

### 2.3. Surface Conditioning

After thermal cycling the composite specimens were sandblasted with aluminum oxide particles (Al_2_O_3_, 50 µm, RondoFlex Preparation Powder; KaVo, Biberach, Germany; 2.5 bar) for 10 s at a distance of 10 mm perpendicular to the composite surface. Loose Al_2_O_3_-particles were removed using an air blower. Sandblasting was followed by 60 s silanization (Monobond Plus, Ivoclar, Schaan, Liechtenstein) and the application of an adhesive (OptiBond FL Adhesive, Kerr, Orange, CA, USA) for 15 s. Light-curing of the adhesive was performed for 15 s at 1200 mW/cm^2^ (Bluephase G2, Ivoclar, Schaan, Liechtenstein).

### 2.4. Repair Restoration

The universal resin composite Ceram.x Spectra ST (Dentsply Sirona, Konstanz, Germany; shade A4) was used as repair composite. The repair composite was adhered onto the composite substrate surfaces in three increments of 1.5 mm thickness each using custom-made cylindrical Teflon molds (diameter 10 mm). The surface of the repair composite increments was flatted using conventional modeling instruments prior to light-curing for 20 s at 1200 mW/cm^2^ (Bluephase G2, Ivoclar, Schaan, Liechtenstein). After completion of the repair restorations, all specimens were submitted to an additional thermal cycling process in artificial saliva (5000 cycles between 5 °C and 55 °C; dwell time: 20 s; transfer time: 15 s) [24].

### 2.5. Micro-Tensile Bond Strength Test

With the aid of a precision cutting machine (Accutom-50, Stuers, Denmark) and a diamond-coated saw wheel (M1D10; Struers GmbH, Ballerup, Denmark), specimens were cut longitudinally in two directions. From each restored specimen, the central nine rectangular sticks of approximately 1 mm × 1 mm × 9 mm were marked and cut parallel to the surface of the SEM carrier using a low-speed precision cutter (IsoMet, Buehler, Lake Bluff, IL, USA) resulting in a total of 72 sticks per group. To calculate the bonding area of each stick, the exact dimensions of the edge lengths of the sticks were measured using a digital caliper (Kisling, Zürich, Switzerland).

The sticks were prepared for micro-tensile bond strength (µTBS) testing by fixing their ends into µTBS jigs (Wenka, Karl Wenger SA, Courgenay, Switzerland) with cyanoacrylate glue (Superglue No. 1733 2000, Renfert GmbH, Hilzingen, Germany). The µTBS jigs had been previously sandblasted (110 mm Al_2_O_3_, 4.5 bar) to enhance adhesion to the sticks.

The sticks were mounted in a universal testing machine (ZwickRoell Z010, ZwickRoell GmbH, Ulm, Germany). Using a load cell of 500 N, a tensile force test was applied at a speed of 1 mm/min until failure occurred. The recorded load (N) when sticks failed divided by the calculated bonding area (mm^2^) resulted in the µTBS (MPa). This value was recorded for each stick.

### 2.6. Failure Mode Analysis

Failure mode analysis was performed with a stereomicroscope (M3B, Wild, Heerbrugg, Switzerland; 15× magnification). Failure types were categorized as cohesive (within the substrate composite or within the repair composite), adhesive (between the substrate and the repair composite), or mixed failure (involving both the interface and either composite material). Failures that occurred within the jigs or involved glue were considered invalid and excluded from the analysis [25].

### 2.7. Statistical Analysis

The µTBS of sticks which failed prior to testing (pre-test failures) was set to 0 MPa [25]. Bond strength values of individual sticks were averaged for each specimen and the obtained mean values were used as statistical units (*n* = 8 per experimental group). Normality of the data distribution was tested using the Shapiro–Wilk test and inspection of normal Q-Q plots. Except for a single extreme outlier in the Ceram group, data did not deviate significantly from the normal distribution. Therefore, the statistical analysis was conducted using a parametric approach (one-way ANOVA), excluding the aforementioned extreme outlier. The extreme outlier was identified according to a predefined and commonly used statistical criterion, namely as a value more than 3.0 × interquartile range (IQR) below Q1 or above Q3. The decision to exclude this value was made to preserve the validity of the parametric assumptions of the one-way ANOVA, particularly the assumption of normality and homogeneity of variance. Multiple comparisons among the materials were performed using Tukey post hoc adjustment at an overall significance level of 0.05. Frequencies of failure modes (adhesive, cohesive in repair material, cohesive in substrate, and mixed) were statistically compared among materials using a chi-squared test. Following a significant result of the omnibus test, pairwise comparisons of proportions were performed using z-tests with Bonferroni adjustment for multiple comparisons. The statistical analysis was made in SPSS (version 25; IBM, Armonk, NY, USA).

## 3. Results

### 3.1. Micro-Tensile Bond Strength

**Figure 2:**
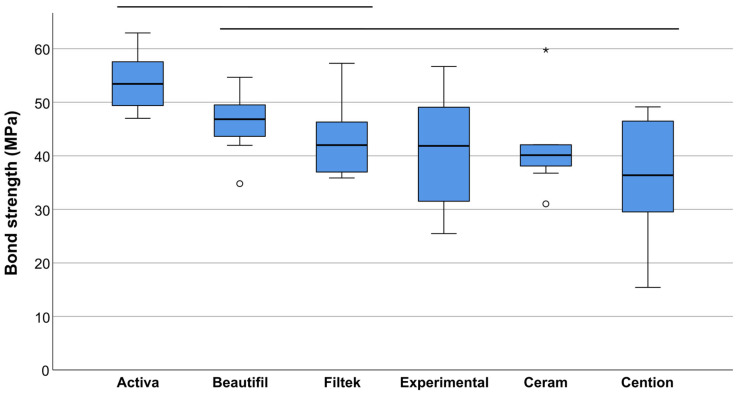
Micro-tensile bond strength (in MPa) of ion-releasing (ACTIVA BioACTIVE-RESTORATIVE, Cention Forte, Beautifil II, experimental composite) and conventional (Ceram.x Spectra ST, 3M Filtek Supreme XTE) resin composites repaired with universal composite material (Ceram.x Spectra ST). Values under the same line show statistically similar results (*p* > 0.05). The boxplots show the medians (bold black lines) with 25% and 75% quartiles (boxes), while the whiskers represent 1.5 × interquartile range (IQR), or minima and maxima of the distribution if below 1.5 × IQR. Outliers are shown as circles and extreme outliers as asterisks.

Figure 2 illustrates the micro-tensile repair bond strengths of all tested groups. The highest mean bond strength values were obtained for Activa, Beautifil, and Filtek (53.8, 46.2, and 43.0 MPa, respectively), which did not differ significantly among each other (*p* = 0.163). With the exception of Activa as the material with the highest bond strength, all other materials showed statistically similar bond strength (*p* = 0.106), with mean values ranging from 36.2 to 46.2 MPa. Statistically significant differences were found in the pairwise comparisons between Activa and each of the three composites with the lowest bond strength values, namely Experimental (*p* = 0.029), Ceram (*p* = 0.010), and Cention (*p* = 0.001). Pairwise comparisons showing mean differences, Cohen’s d, and 95% confidence intervals are given in Table 2.

### 3.2. Failure Mode Analysis

**Figure 3:**
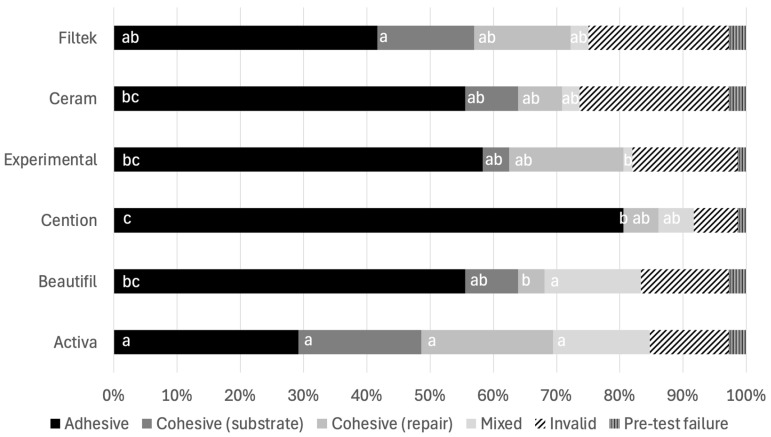
Percentage (%) of failure mode distribution per group. Within each failure mode, same letters indicate statistically similar values (*p* > 0.05).

The distribution of the failure modes within the six groups is shown in Figure 3. Adhesive failure was the predominant failure mode of all groups, with a percentage distribution ranging from 29.17% (Activa) to 80.56% (Cention). Activa showed the highest incidence of cohesive failures (substrate and repair composite) among the groups.

## 4. Discussion

The results of the present in vitro study revealed significant differences in the repair bond strength between composite materials. Therefore, the null hypothesis was rejected.

Common reasons necessitating repair restorations include fractures and secondary caries, which imply that repairs are generally needed only after a certain period of clinical service [1,2,26,27,28]. To simulate the aging process, specimens in this study were artificially aged following the protocol of Wiegand et al. [24]. Prolonged water or saliva storage was omitted, potentially limiting water sorption [29]. Bond strength values recorded in this study ranged from 36.2 to 53.8 MPa, which exceeded those in other studies with additional water storage [30], likely due to reduced degradation [31,32]. Water absorption leads to hydrolytic degradation at the resin–filler interface, leading to a reduced number of unsaturated double bonds able to react with the repair composite [30]. By omitting additional water storage, hydrolytic degradation was likely limited, which may have masked potential material-dependent differences in repair bond strength.

Following Burrer et al. [33], in the present study, sandblasting with aluminum oxide was performed at a 10 mm distance to the substrate surface. This distance is recommended for optimal interlocking between substrate and repair composite material, as larger distances increase particle scattering, enabling a more homogenous roughening [33]. This, in turn, increases the surface area and creates a microretentive bonding pattern, facilitating improved penetration of the silane coupling agent and adhesive [34,35,36]. Since micromechanical interlocking represents the main bonding mechanism in composite repair [36,37,38], these surface characteristics are particularly relevant.

The surface characteristics were intentionally influenced by sandblasting and were additionally influenced indirectly by degradation induced during thermocycling [39]. Depending on the material and its composition, artificial aging can show varying effects on surface roughness. A study investigating different ion-releasing resin composites stored in artificial saliva concluded that increased concentrations of calcium and phosphate indicated that giomers and glass ionomers displayed eroded surfaces with a loss of filler particles, as evidenced by SEM images [40]. Cention Forte exhibited a significantly higher release of calcium and fluoride compared to the other tested materials, including Beautifil II [40]. Our results showed no significant repair bond strength differences between Cention and Beautifil, likely due to limited water sorption masking any potential differences. The comparatively high µTBS values in this study compared to other studies with prolonged water storage [30] support this observation.

Carneiro et al. [16] examined the surface roughness of Activa BioACTIVE-Restorative and Filtek Supreme XTE before and after thermocycling. The results revealed a large dispersion between specimens, which increased after thermocycling. Statistically significant differences in surface roughness measurements were also found between Activa BioACTIVE-Restorative and Filtek Supreme XTE before and after aging. Filtek Supreme XTE demonstrated the most stable values, experiencing the least change due to aging. In the present study, artificial aging may have increased the surface roughness of Activa to a level that enhanced its repair bond strength, possibly due to improved infiltration of the bonding systems into the roughened surface, while especially the conventional resin composites were stable. A limitation of this study is that no roughness measurements or SEM images were taken to confirm this assumption, leaving it speculative.

In this study, Monobond Plus was used as the silane coupling agent to improve wettability of the substrate composite and facilitate infiltration of the bonding agent into the microretentive substrate surface [41,42,43]. Following application of silane, the adhesive system was applied. In this case, only the adhesive from the two-step adhesive system OptiBond FL was utilized. In clinical practice, it is nearly impossible to treat only the composite or dentin, meaning that some primer will inevitably come into contact with the composite. Rathke et al. [44] found that in the case of a two-step application, the bonding forces are equal to or greater than those observed when primer is not applied. This finding suggests that the use of a multi-stage bonding system makes it possible to limit the application of primer primarily to cases where exposed dentin is present at the repair site [44].

The ion-releasing materials utilized in this study are predominantly hydrophilic, whereas the conventional resin composites are classified as hydrophobic. Hydrophilic materials facilitate opportunities for hydrogen bonding with hydroxyethyl methacrylate (HEMA) [45], a component of the adhesive, as well as dipole–dipole interactions with the hydrophobic adhesive. These interactions are absent when hydrophobic materials are repaired with other hydrophobic counterparts. In addition, hydrophilic materials have higher surface energy and thus improved wettability [46]. This may contribute to relatively high repair bond strength values, comparable to or even exceeding those of hydrophobic conventional resin composites. All materials (Beautifil; Ceram; Cention; Experimental; Filtek) showed statistically similar bond strength, except for Activa, which is formulated with a patented ionic resin (Embrace resin) that contains a small amount of water [47]. The high repair bond strength values suggest that Activa is the most hydrophilic of all ion-releasing materials. This finding is consistent with its classification as a resin-modified glass ionomer cement [48], which is, amongst others, characterized by the presence of water in its formulation and hydrophilic behavior [48,49].

Activa also is the only material used which is classified as flowable. Due to their low elastic modulus and high flexural strength, flowable composites are known to better absorb stress compared to condensable materials [50]. This is consistent with the findings of Carneiro et al. [16] and may further explain the high mean repair bond strength values observed for Activa.

Remarkably, Activa not only exhibited the highest repair bond strength values, but also the highest proportion of cohesive fractures (40%), while the other groups exhibited predominantly adhesive failures. Cohesive fractures can occur if there is a particularly strong adhesive bond at the repair site or if there is a degradation of the substrate material due to aging processes. In the present study, the high bond strength values and the even distribution of cohesive fractures in both the substrate and the repair composite indicate a strong adhesive interface between Activa and the repair composite. This observation is in line with the findings of Ozaslan et al. [21], where groups with low µTBS values generally exhibited adhesive failures, while groups with high µTBS values showed a higher proportion of cohesive and mixed failures. The same conclusion was also reached by Awad et al. [20] in their study.

We assume that the overall outcome for Activa observed in our study is likely the result of the combined influence of material classification, hydrophilicity, and flowable behavior. More detailed investigations would be necessary to determine the specific contribution of each individual factor to its performance.

The experimental composite was based on a conventional Bis-GMA/TEGDMA resin matrix with a total filler load of 70 wt% (50 wt% silanized reinforcing fillers and 20 wt% unsilanized bioactive glass). Because the bioactive glass is not silanized and therefore cannot form covalent bonds with the resin matrix, and because it gradually dissolves in an aqueous environment, it can be expected to reduce the cohesive strength of the material and impair repair bond strength. In this context, it is noteworthy that the experimental composite performed similarly to established commercial composites with respect to bond strength. Moreover, it exhibited a statistically similar fraction of cohesive failures compared with the commercial composites, indicating that the presence of soluble unsilanized particles was not the limiting factor for composite cohesive strength.

A limitation of this in vitro study is that no positive reference groups were included, which ideally match the inherent strength [51] and represent the maximal repair potential in relation to the cohesive strength of the materials tested [43]. Furthermore, the repair was only carried out with one repair protocol, which may have contributed to potential ceiling effects. Therefore, the results cannot be generally applied to other adhesive systems and composites, as different coupling agents, bonding agents and repair materials may lead to different results. In addition, prolonged water or saliva storage was omitted, potentially limiting hydrolytic degradation and water sorption, which may have masked material-dependent differences in repair bond strength and could have contributed to the comparatively high bond strength values observed. Furthermore, surface characterization was not performed, which would have provided a more objective explanation for the differences in µTBS values. Future studies should evaluate different aging strategies, multiple repair systems, and clinically relevant contamination scenarios, as well as correlate bond strength results with surface and chemical analyses. Including fatigue or cyclic loading tests would also improve clinical relevance.

## 5. Conclusions

Within the limitations of this in vitro study, the results of the present study indicate that ion-releasing resin composites can be effectively repaired with conventional universal composite and show repair bond strength values at least as high as those of conventional composite materials. Activa exhibited the highest bond strength values and the greatest proportion of cohesive failures, indicating a strong adhesive interface with the repair composite, while showing no significant difference in bond strength compared with Beautifil and Filtek.

## Figures and Tables

**Table 1:** Composition of the composite materials used in the present study.

Product (Code)	Shade Lot Number	Composition	Filler Load	Manufacturer
ACTIVA BioACTIVE- RESTORATIVE (Activa)	A2 211117	**Matrix:** Modified diurethane, other methacrylates monomers, modified polyacrylic acid (∼53.2%) **Filler:** Silica (∼3.0%), amorphous, sodium fluoride (∼0.9%)	56 wt% 21.6% reactive ionomer glass fillers	Pulpdent, Watertown, MA, USA
Beautifil II (Beautifil)	A2 062295	**Matrix:** Bis-GMA, TEGDMA **Filler:** S-PRG glass filler, fluoride-containing fluoro-boro-alumino silicate glass filler particles, polymerization initiator, pigments	83.3 wt% 69 vol%	SHOFU DENTAL, Kyoto, Japan
Cention Forte (Cention)	A2 Z03VK3	**Matrix:** UDMA, aromatic aliphatic UDMA, DCP, PEG-400-DMA, hydroperoxide, ivocerin, acyl phosphine oxide **Filler**: Inert barium alumino-boro-silicate glass, ytterbium fluoride, calcium fluoro-alumino-silicate glass, SiO_2_-CaO-CaF_2_-Na_2_O glass	58–59 vol% 78.4 wt%	Ivoclar, Schaan, Liechtenstein
Experimental Composite (Experimental)	N/A N/A	**Matrix:** Bis-GMA (59.4 wt%), TEGDMA (39.6 wt%), camphorquinone (0.2 wt%), amine (0.8 wt%) **Filler:** 20 wt% experimental fluoride-containing bioactive glass (particle size (d50) ≈ 3 μm, therefrom: SiO_2_ (33.5 wt%), CaO (33.0 wt%), Na_2_O (10.5 wt%); P_2_O_5_ (11.0 wt%); CaF_2_ (12.0 wt%))	20 wt% experimental fluoride-containing bioactive glass; 50 wt% reinforcing fillers: Inert barium glass:silica, 2:1	N/A (prepared in-house)
Ceram.x Spectra ST (Ceram)	A2 2010000546 A4 2207000292	**Matrix:** Methacrylic modified polysiloxane nanoparticles, dimethacrylate resin, ethyl-4-(dimethylamino)benzoate **Filler:** Spherical, pre-polymerized SphereTEC fillers (particle size ≈ 15 μm), non-agglomerated barium glass, CQ, ytterbium fluoride	78–80 wt% 60–62 vol%	Dentsply Sirona, Konstanz, Germany
3M Filtek Supreme XTE (Filtek)	A2 NF44127	**Matrix:** Bis-GMA, PEGDMA, TEGDMA, UDMA **Filler:** Non-agglomerated/non-aggregated silica (20 nm) and zirconia (4–11 nm) fillers, aggregated zirconia/silica cluster filler (average cluster particle size: 0.6–10 nm)	78.5 wt% 63.3 vol%	3M Oral Care, St. Paul, MN, USA

Bis-GMA: Bisphenol A-glycidyl methacrylate; TEGDMA: Triethylene glycol dimethacrylate; S-PRG: Surface pre-reacted glass ionomer; UDMA: Urethane dimethacrylate; DCP: Dicalcium phosphate; PEG-400DMA: Polyethylene glycol 400 dimethacrylate; CQ: Camphorquinone; PEGDMA: Polyethylene glycol dimethacrylate.

**Table 2:** Pairwise comparisons showing mean differences, Cohen’s d, and 95% confidence intervals.

Comparison	Cohen’s d	Mean Difference	95% Confidence Interval
Activa–Beautifil	0.95	7.66	−4.42–19.73
Activa–Cention	2.19	17.67	5.60–29.74
Activa–Ceram	1.80	15.04	2.54–27.53
Activa–Experimental	1.60	12.95	0.88–25.02
Activa–Filtek	1.35	10.87	−1.20–22.95
Beautifil–Cention	1.24	10.01	−2.06–22.09
Beautifil–Ceram	0.88	7.38	−5.11–19.88
Beautifil–Experimental	0.66	5.29	−6.78–17.37
Beautifil–Filtek	0.40	3.22	−8.85–15.29
Cention–Ceram	0.31	2.63	−15.13–9.86
Cention–Experimental	0.58	4.72	−16.79–7.35
Cention–Filtek	0.84	6.8	−18.87–5.28
Ceram–Experimental	0.25	2.08	−14.58–10.41
Ceram–Filtekl	0.50	4.16	−16.66–8.33
Filtek–Experimental	0.26	2.08	−9.99–14.15

## Data Availability

The original contributions presented in this study are included in the article. Further inquiries can be directed to the corresponding author.
